# Parental variables associated with risk for Borderline Personality Disorder Development in adolescence: a scoping review

**DOI:** 10.1192/j.eurpsy.2026.11910

**Published:** 2026-07-31

**Authors:** F. Rospo, M. Tironi, S. Charpentier Mora

**Affiliations:** Department of Educational Sciences, University of Genoa, Genoa, Italy

## Abstract

**Introduction:**

Adolescence is the first developmental period in which Borderline Personality Disorder (BPD) features (i.e., instability in identity, affectivity, and relationships) begin to manifest. This phase is therefore critical for understanding the dynamics that may lead to personality pathology, given its association with high comorbidity and reduced quality of life. Although literature highlights the role of maladaptive parental factors - psychopathology, dysfunctional parenting - most of the empirical evidence comes from retrospective adult samples. Research directly examining parental influences during adolescence remains limited, underscoring the need to map the current literature to identify gaps and guide future studies.

**Objectives:**

This scoping review maps and synthesizes the literature on parent-realed variables associated with adolescent BPD.

**Methods:**

This study followed PRISMA-ScR guidelines. Searches of major psychological and medical databases were conducted up to March 31, 2025. Records were included if published in English, involved adolescents with BPD features (ages 11–19), and examined parent-related variables.

**Results:**

Of 4,922 records, 70 articles met inclusion criteria. Within this final pool, several studies assessed multiple parent-related variables concurrently. Parenting variables were examined in 43 studies, showing lower parenting capacities in families of adolescents with BPD, though positive parenting behaviors were protective. Maladaptive practices - invalidation, psychological control, overprotection - were the most frequently studied and significant risk factors. 29 studies reported parental psychopathology associated with higher adolescent BPD scores both cross-sectionally and prospectively. Parent-to-child maltreatment and family dysfunction were also identified as risk factors. Two studies showed that parents’ own childhood adversity and low-quality parenting increased adolescent BPD risk. Finally, regarding other theoretically and clinically relevant individual variables, two studies found that parental mentalization was not directly associated with adolescent BPD, whereas two other studies suggested that maternal emotion regulation difficulties may contribute to the intergenerational transmission of BPD by indirectly linking mothers’ BPD to adolescents’ BPD through both maternal and adolescent difficulties.

**Conclusions:**

Key findings highlight the critical role of parental variables in adolescent BPD. Parental psychopathology, reduced positive parenting, maladaptive practices, and parent-to-child maltreatment emerged as key risk factors. Few studies examined clinically and theoretically relevant individual parental variables (e.g., mentalization), which may contribute to vulnerability transmission. The findings underscore the clinical importance of family-focused research and the need to investigate the psychological mechanisms underlying BPD risk.

**Disclosure of Interest:**

None Declared

